# Graphene and novel graphitic ZnO and ZnS nanofilms: the energy landscape, non-stoichiometry and water dissociation†

**DOI:** 10.1039/c8na00155c

**Published:** 2019-04-01

**Authors:** Sergio Conejeros, Neil L. Allan, Frederik Claeyssens, Judy N. Hart

**Affiliations:** School of Chemistry, University of Bristol Cantock's Close Bristol BS8 1TS UK neil.allan@bristol.ac.uk; Departamento de Química, Universidad Católica del Norte Av. Angamos 0610 Antofagasta 1240000 Chile; University of Sheffield, Department of Materials Science and Engineering, Kroto Research Institute Broad Lane Sheffield S3 7HQ UK; School of Materials Science & Engineering, UNSW Sydney NSW 2052 Australia

## Abstract

The energy landscapes of ultra-thin nanofilms of ZnO and ZnS are examined in detail using periodic hybrid density functional calculations. We predict new staggered graphitic forms, which are stable only for the thinnest films and are of particular interest as the electronic structure shows a spontaneous symmetry breaking across the film and consequently a marked decrease in band gap with thickness. The relative energies of the various forms, their structural and electronic properties and their variation with film thickness are discussed. Possible kinetic pathways for transitions from the graphitic forms are examined by explicit evaluation of transition state energies. For polar surfaces, such as (0001) würtzite and (111) zinc blende, many different mechanisms operate to remove or reduce the surface dipole depending on the number of layers in the nanofilm. The polar ZnS nanofilms, but not the polar ZnO analogues or any non-polar film, are predicted to spontaneously become non-stoichiometric by loss of zinc atoms from the surface. The behaviour of adsorbed water on the ultra-thin films is also examined. There is no dissociation on any ZnS film. For ZnO, dissociation into OH^−^ and H^+^ takes place not only on (101̄0) würtzite, but also on (110) zinc blende. This result that does not appear to have been reported previously and deserves future experimental study. While we concentrate on ZnO and ZnS, similar energy landscapes are expected for any oxide or sulphide which adopts the würtzite or zinc blende structure in the bulk.

## Abstract 2

*Ab initio* energy landscapes of thin ZnO and ZnS films reveal new structures, non-stoichiometry and different behaviour of adsorbed water.

## Graphical abstract

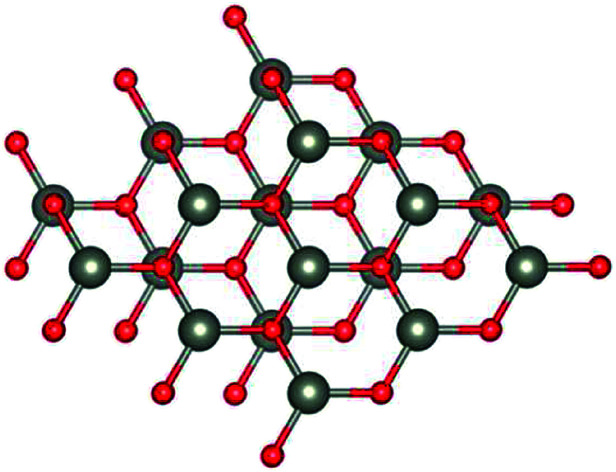

## Introduction

**Fig. 1:**
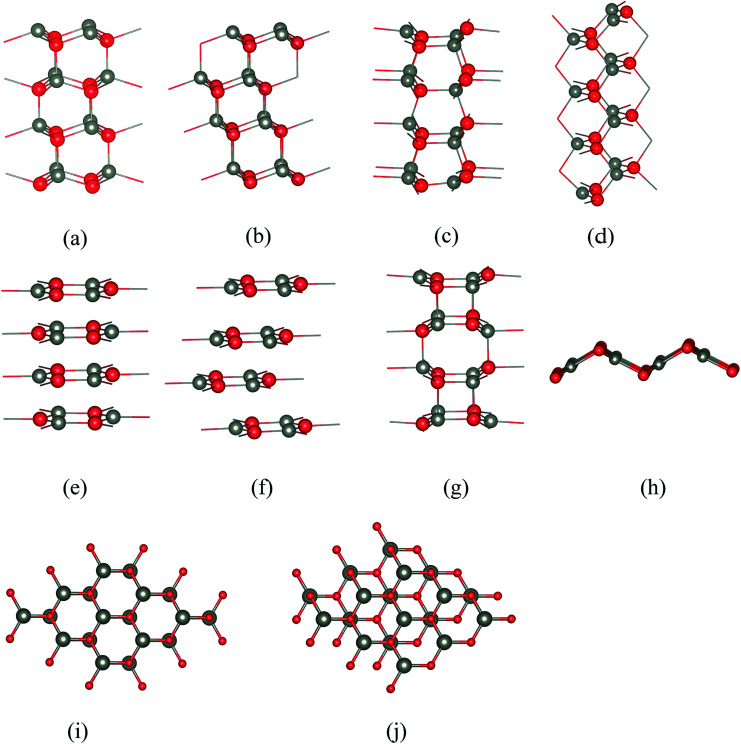
Stacking sequences of the various structures considered in this work: (a) polar wurtzite WZ(0001), (b) polar zinc blende ZB(111), (c) non-polar WZ(101̄0), (d) non-polar ZB(110), (e) “eclipsed” graphitic in which all ions in one layer lie directly above ions of the opposite charge in the layer below, (f) “staggered” graphitic in which adjacent layers are displaced relative to each other, (g) BCT showing the network of quadrilaterals and octagons in the stacking sequence, and (h) V structure. (i and j) Top views of the (i) eclipsed and (j) staggered conformations. Cations are grey and anions red.

Thin films often have structures and properties which differ substantially from those of the bulk. Enormous effort is for example being paid to non-carbon graphene-like two-dimensional nanomaterials. Thin films of ionic and semi-ionic solids provide unrivalled opportunities for the experimental characterisation and fabrication of polar nanostructures with unusual physical and chemical properties and applications in fields as diverse as catalysis^1^ and spintronics.^2^ Of particular significance is a subgroup of such films which are terminated by so-called ‘polar’ (Tasker Type III) surfaces.^3^ Here the stacking sequence involves layers of ions such that the repeat unit has a non-zero dipole moment perpendicular to the surface (*e.g.*, Fig. 1a and b) and the resulting divergent electrostatic contribution to the energy leads to intrinsic instability. Where such surfaces are observed, the macroscopic dipole is reduced^4–6^ or removed. Several possible mechanisms for this stabilisation have been proposed and observed experimentally,^7^ including adsorption of hydrogen,^8^ vacancy formation,^9^ massive surface reconstructions,^10^ and charge transfer from the anion to the cation surface with a corresponding change in electronic structure, such as noted for ZnO.^11^

Both bulk ZnO and ZnS can crystallise either in a hexagonal würtzite-type (WZ) or a cubic zinc blende-type (ZB) structure. The würtzite structure is the most stable for ZnO, and zinc blende for ZnS. In both of these structures, there are polar surfaces that play a key role in film growth, (0001)/(0001̄) for würtzite and (111) for zinc blende. Since the orientation of polar surfaces is such that each repeat unit in the direction perpendicular to the interface has a non-zero dipole moment, they often display behaviour strikingly different from non-polar surfaces and so are the subject of intense current interest.

In previous work we examined polar (0001)/(0001̄) nanofilms of ZnO and other würtzite materials and demonstrated a novel mechanism for removal of the dipole.^12^ We predicted, prior to experimental verification by X-ray diffraction, scanning tunnelling microscopy (STM) and transmission electronic microscopy (TEM),^13–15^ a transition to a hexagonal graphitic-like structure. In this graphitic structure, referred to as “eclipsed”, Zn atoms in one layer lie directly above the O atoms in the next. We found that ultra-thin (0001)/(0001̄) films of würtzite AlN, BeO, GaN, SiC and ZnS also form this graphitic-like structure and have discussed the possible implications of this for the dominance of the morphology of crystalline ZnO by the (0001) and (0001̄) surfaces.^12^

Subsequently, for ZnO, a BCT (Body Centred Tetragonal) structure has been predicted as a new stable phase in free-standing thin films, lower in energy than both the eclipsed graphitic and polar (0001)/(0001̄) structures for certain film thicknesses.^16–18^ This structure has been also suggested theoretically for ZnO nanocrystals^19,20^ and nanorods under tensile strain.^21^ The BCT structure has also been observed experimentally in ZnS nanocrystals grown by chemical co-precipitation^22^ and in the outermost reconstructed layers of single-crystalline (101̄0) ZnO nano-islands.^23^ However, somewhat surprisingly we have found no experimental reports of this structure in thin films.

In this paper, for the first time we examine structures based on that of zinc blende. We compare the stabilities of polar and non-polar structures for different film thicknesses and investigate the energy barriers between different structures. The energy landscapes turn out to be considerably more complex than anticipated and we discuss in detail why this is so, concentrating on the large number of different mechanisms that may remove or reduce surface dipoles. We consider first total energies and optimised structures as a function of film thickness, and then move onto the electronic properties for which hybrid density functional methods have been shown to be much superior to the more traditional LDA (local density approximation) or GGA (generalised gradient approximation) methods. We investigate the kinetic stability of the eclipsed, staggered and BCT structures and possible mechanisms for their interconversion. Water absorption on the different surfaces is also examined. Finally, we summarise the predictions we make in this work and comment on the general applicability of our conclusions.

## Surface structures

Five of the thin film structures we consider are WZ(101̄0), ZB(110), and BCT, which have non-polar surfaces, as well as WZ(0001) and ZB(111), which have polar surfaces (Fig. 1). There are two additional structures with different stackings of graphene layers. The first of these we denote “eclipsed” (Fig. 1e) because all ions in one layer lie directly above those in the adjacent layer; the second we denote “staggered” because adjacent layers are displaced relative to each other as shown in Fig. 1f.

All seven structures contain layers of six-membered Zn_3_X_3_ (X = O, S) rings parallel to the surface,^16^ and in this paper we use the word “layer” to denote a layer of such rings rather than to refer to separate layers of cations and anions. In the polar WZ and ZB structures, these six-membered rings have chair conformations. The rings in the non-polar WZ(101̄0) structure (Fig. 1c) are non-planar with boat conformations (*cf.* cyclohexane) in the layers parallel to the surface. Perpendicular to this surface is a stacking sequence of six-membered rings in chair conformations. The non-polar ZB(110) structure (Fig. 1d) has similar rings all in chair conformations both in layers parallel and perpendicular to the surface. The BCT structure (Fig. 1g) contains a network of quadrilaterals and octagons along the direction perpendicular to the surfaces of the film. All the six-membered rings in this structure have boat conformations; in adjacent layers in the stacking sequence perpendicular to the surface the rings are oriented back-to-back, in contrast to the non-polar WZ(101̄0) structure. This gives rise to the characteristic four- and eight-membered rings connecting the layers in the BCT structure. In the graphitic structures, the six-membered rings are planar or almost so. In contrast to the other structures, these flat rings have three-fold trigonal planar (rather than four-fold tetrahedral) coordination, and larger X–Zn–X bond angles.

## Computational methodology

Calculations, periodic in two dimensions, were performed for different slab thicknesses. For each of the seven crystal structures, slab thicknesses of 1–12 layers were considered. Both the primitive cell and an appropriately oriented 2 × 2 surface supercell expansion were used for WZ(0001), ZB(111), eclipsed and staggered graphitic structures; a 2 × 1 surface supercell was used for ZB(110) and WZ(101̄0). No atoms are held fixed.

Calculations were performed using the *ab initio* CRYSTAL14 code^24–26^ to evaluate the relative energies and electronic properties. No spurious 3D periodicity is required for low-dimensional systems as when a plane-wave basis set is used.^24^ Energies were calculated using periodic Density Functional Theory (DFT) adopting the hybrid B3LYP functional^27^ and Grimme's^28^ dispersion correction as implemented in the CRYSTAL14 program. All-electron atomic Gaussian basis sets were used. The basis sets used were 86-4111(d41G), 8-411G, and 86-311G, optimized for Zn^2+^, O^2−^, and S^2−^, respectively.^29–31^ For the calculation of the Coulomb and exchange integrals, tolerance factors of 7, 7, 7, 7, and 14 were used. The convergence criterion for the electronic energy was set at 10^−7^ a.u.^32^ For supercell calculations, the reciprocal space integration used a mesh of 6 × 6 × 1 *k*-points in the irreducible Brillouin zone chosen according to the Monkhorst–Pack scheme;^33^ the convergence of the energy with the grid size was checked. Our geometry optimisations relaxed all degrees of freedom. The calculated lattice parameters and band gaps for ZnO and ZnS are in good agreement with the experimental values (Table 1) where data are available.

**Table:** Calculated and experimental cell parameters and band gaps for ZnO and ZnS

	Structural parameters				Band gap	
	*a* (Å)		*c* (Å)		*E*_g_ (eV)	
	Calc.	Exp.	Calc.	Exp.	Calc.	Exp.
**Wurzite structure**						
ZnO	3.256	3.250	5.234	5.207	3.29	3.44 (ref. 34)
ZnS	3.838	3.820	6.269	6.260	3.91	3.77 (ref. 35)

**Zinc blende structure**						
ZnO	4.582	4.580	—	—	3.10	3.27 (ref. 36)
ZnS	5.426	5.410	—	—	3.89	3.72 (ref. 37)

## Results and discussion

### A. Structural stability of the ZnO and ZnS surfaces

#### ZnO

**Fig. 2:**
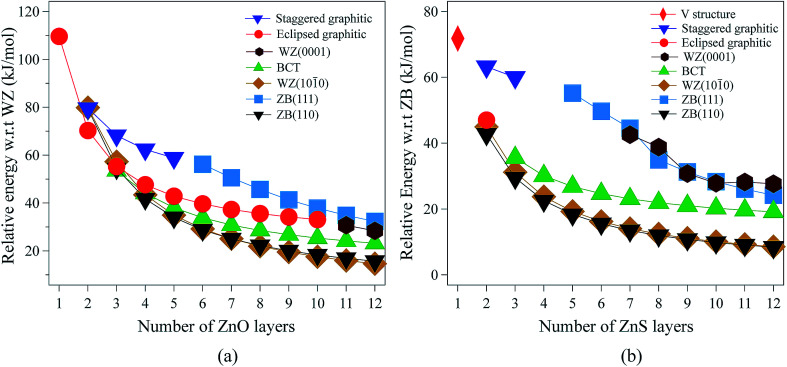
Energies (per formula unit) for (a) ZnO and (b) ZnS nanofilms. Energies are relative to the lowest energy bulk phase, *i.e.*, the würtzite and zinc blende phases for ZnO and ZnS, respectively.

The relative energies in Fig. 2a confirm that, for one layer, the graphene structure is lowest in energy for ZnO, in agreement with previous work.^11,12,38^ For 2 layers, the eclipsed graphitic form (Fig. 1e) is lowest in energy, consistent with previous reports for 2 and 3 layers.^11,12,16,18^ In this eclipsed graphitic structure, all the six-membered rings are regular hexagons irrespective of slab thickness, and all Zn–O–Zn bond angles within each layer are 120°. The Zn–O interatomic distance within each layer is smaller (∼2%) than that in bulk würtzite. In contrast, the Zn–O distance between the layers in the eclipsed graphitic form is substantially larger (∼20%) than that between the layers in bulk würtzite, consistent with the change from tetrahedral to trigonal coordination.

For 3 layers, the non-polar BCT structure (Fig. 1g) is lowest in energy. With further increase in the number of layers, first the non-polar ZB(110) structure (Fig. 1d) (from 4 to 7 layers), and then the non-polar WZ(101̄0) film (Fig. 1c) (from 8 to 12 layers) become the most stable.

Polar WZ(0001) films up to 10 layers thick, and with a primitive surface cell, relax during the optimisation to the eclipsed graphitic structure and so energies for the WZ(0001) films are plotted in Fig. 2a only for 11 and 12 layers. In similar calculations, but using a 2 × 2 surface supercell, such films up to only 4 layers thick automatically relax to the eclipsed graphitic structure, and film thicknesses of 5 and 6 layers relax to BCT. Even in the thickest polar WZ(0001) films (11 and 12 layers, Fig. 1a), which are kinetically stable, a flattened graphitic-like structure emerges in the outermost layer with the bulk tetrahedral coordination with formally sp^3^-hybridised oxygen changing to a flattened structure with sp^2^-hybridised oxygen. Similar to the eclipsed graphitic structure, the Zn–O distances within these top and bottom layers are 1.9% and 3.0%, respectively, less than in the bulk and the surface bond angle (O–Zn–O) is almost hexagonal, at approximately 115° and 117°, respectively. The interior of the polar WZ(0001) slabs remain more bulk-like with Zn–O distances just 0.8% smaller than the bulk value. The average bond angle in the slab interior (O–Zn–O) is approximately 113.7°.

In the non-polar WZ(101̄0) films, significant changes in the interatomic distances relative to bulk ZnO (>1%) are limited to the three uppermost layers; consistent with the detailed discussion in ref. 11.

The staggered graphitic (Fig. 1f) film is not the lowest in energy for any film thickness. However, when the number of ZnO bilayers is fewer than 6, it is a local minimum in the energy landscape and the polar ZB(111) films relax spontaneously to this structure. The interatomic distances within the hexagonal layers in the staggered graphitic form are ∼4% less than in bulk würtzite, and ∼1.5% less than in the eclipsed graphitic form. In contrast, the interlayer separation is ∼8% larger than in the eclipsed graphitic. As in the eclipsed graphitic structure, the bond angles within each layer are all 120°. For thicknesses greater than six layers, films initially with the staggered graphitic structure optimise spontaneously to the polar ZB(111) structure.

Thus, a rich structural chemistry emerges for thin films of ZnO. There are a large number of local minima in the energy landscape, representing different routes for structural relaxation to reduce or remove the dipole perpendicular to the surface before relaxation, if present. For example, one mechanism is extensive relaxation to non-bulk (*e.g.* graphitic) structures, but there is an associated energy penalty due to altered bond lengths and angles, and this increases with film thickness. Another possibility, discussed below, is charge transfer between layers. In experimental settings, there are various other possibilities that are not considered here, such as absorption of molecular species, formation of stepped surfaces and hydroxylation.^5–7^

#### ZnS

The relative energies of the different film structures for ZnS are shown in Fig. 2b. When there is only one layer, ZnS adopts the V structure, identical to that reported in ref. 38, which is a buckled non-planar graphene structure (Fig. 1h), with the sulfur atoms lying 0.85 Å above and below the plane of the zinc atoms.

The eclipsed graphitic structure is only stable for two layers and, unlike the ZnO analogue, there is a marked deviation from planarity, reflecting the reduced preference of sulfur relative to oxygen for planar coordination.^39^ As for ZnO, the interatomic distances within the layers are smaller (1.0%) than in the bulk but the separation between the layers is larger (11.2%). The S–Zn–S bond angles within a layer are 116° consistent with the non-planarity.

From 2 layers onwards, the non-polar ZB(110) and WZ(101̄0) structures are the most stable, with the former slightly lower in energy than the latter, consistent with the relative stabilities of bulk zinc blende and würtzite ZnS.

For thicknesses between 3 and 6 layers, both the eclipsed graphitic and polar WZ(0001) structures spontaneously relax to BCT, in contrast to ZnO. In the outermost layers, the S–Zn–S angles vary from 114° to 123°. Such a change in the outermost layers is not restricted to the BCT structure – in the non-polar ZB(110), WZ(101̄0) and BCT, half of the Zn atoms in the outermost layer have a planar trigonal coordination. In contrast to ZnO, this BCT structure is not the lowest in energy for any film thickness, but it is a local minimum. After 6 layers WZ(0001) becomes kinetically stable and the eclipsed graphitic structure relaxes to WZ(0001).

Similar to ZnO, albeit only up to a thickness of 3 layers, polar ZB(111) films reconstruct to the staggered graphitic structure. Once again, the interatomic distances within the layers are smaller (∼3%) than in bulk zinc blende ZnS and the separation between the layers is larger (∼20%). The bond angles within a layer are around 118°, again indicating a small deviation from planarity, in contrast to ZnO. We observe essentially no variation of the geometry with film thickness up to 3 layers. For film thicknesses greater than 4 layers, the staggered graphitic films become unstable and spontaneously reconstruct to the polar ZB(111) structure. Both the staggered graphitic and ZB(111) films, even though local minima for some film thicknesses, are relatively high in energy.

The ESI† shows the relaxed structures for ZnS which differ significantly after optimisation from the “ideal” structures shown in Fig. 1 – eclipsed graphitic (2 layers), staggered graphitic (3 layers), BCT, ZB(110) and WZ(101̄0). Overall ZnS shows some similarities to ZnO but there are significant differences such as a stronger preference for the non-polar structures.

### B. Formation of non-stoichiometric polar ZnS surfaces

**Fig. 3:**
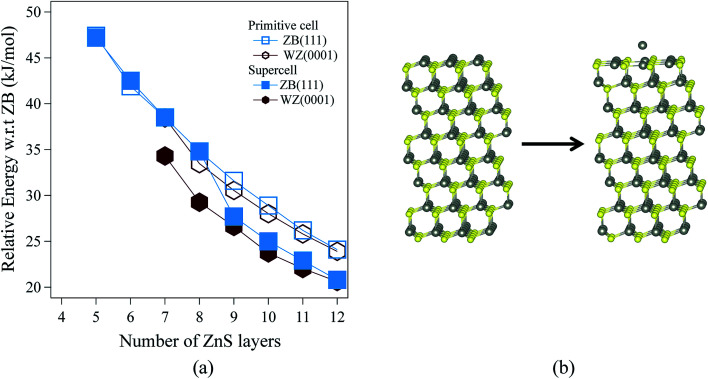
(a) Relative energies for ZnS WZ(0001) and ZB(111) films, based on calculations using both a primitive cell and a surface 2 × 2 supercell. (b) Schematic representation of the initial and the optimised ZnS ZB(111) film showing the surface reconstruction by expulsion of a Zn atom. Zn atoms are grey and S atoms are yellow.

Fig. 3 shows the relative energies of the polar WZ(0001) and ZB(111) films for ZnS. Results are plotted for two different surface cell sizes, one the primitive cell with two atoms per layer (for which results were not shown in Fig. 2b), and the second, as in Fig. 2b, a 2 × 2 surface supercell. The results of these calculations demonstrate the importance of using supercells, since the supercells show a surface reconstruction, with expulsion of a Zn atom from the top layer and formation of a substoichiometric material, for both WZ(0001) and ZB(111) films when they contain more than eight layers. The interatomic distance between the Zn atom that is expelled and the closest S atom in the surface is ∼30% larger than the bulk Zn–S interatomic distance. The atoms remaining in the top layer form a planar sheet. This behaviour is not observed in a calculation on the primitive surface unit cell due to the geometric restriction it imposes. Analogous behaviour is not observed for ZnO. This surface reconstruction has been previously noted for WZ(0001) ZnS films^17^ and we discuss why this takes place later in our discussion of electronic structure.

### C. Electronic properties

Our calculations using hybrid DFT reproduce well the band gaps of both bulk ZnO and ZnS (Table 1). In contrast, it is well known that pure DFT (LDA and GGA) underestimates the band gap of bulk ZnO and ZnS.^40–44^ All the systems studied in this work have direct band gaps. In ZnO and ZnS, the top of the valence band is composed predominantly of anion p states, while the bottom of the conduction band is composed mostly of Zn 4s states.

**Fig. 4:**
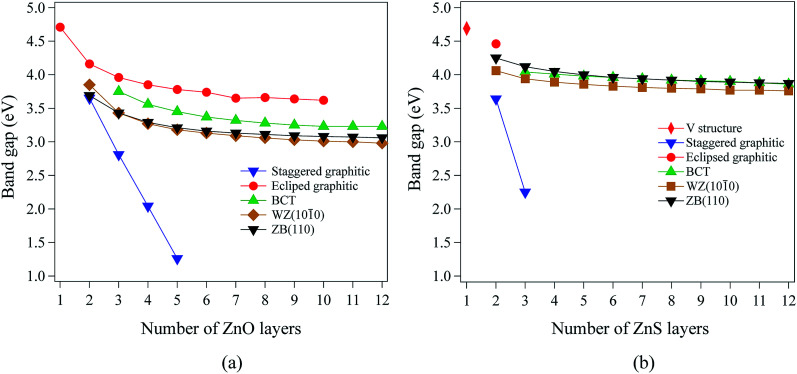
Band gaps as a function of film thickness for non-polar (a) ZnO and (b) ZnS films.

For films with a thickness of one layer, the predicted band gaps for ZnO (graphene) and ZnS (V structure) are 4.70 eV and 4.69 eV, respectively. Fig. 4a and b show that, for the non-polar films, band gaps decrease as thickness increases (due to quantum confinement), tending to values between 3.0 eV and 4.2 eV for ZnO and between 3.7 eV and 4.5 eV for ZnS.

**Fig. 5:**
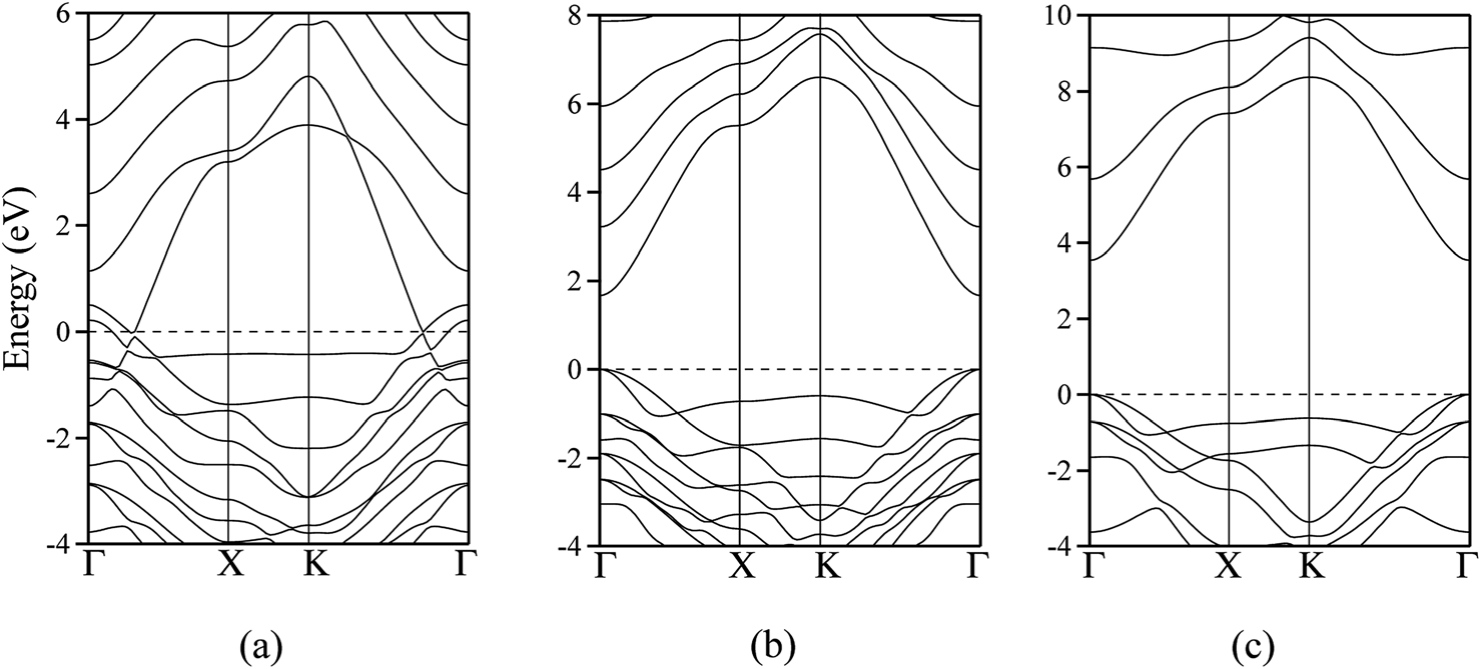
Dispersion curves for ZnO: (a) polar ZB(111) structure with a thickness of 6 layers. (b) staggered graphitic structure with a thickness of 2 layers; (c) staggered graphitic structure with a thickness of 4 layers. The energy of the top of the valence band has been arbitrarily assigned to zero. *Γ* = (0,0), *X* = (a*/2,0) and *K* = (a*/3, a*/3).

**Fig. 6:**
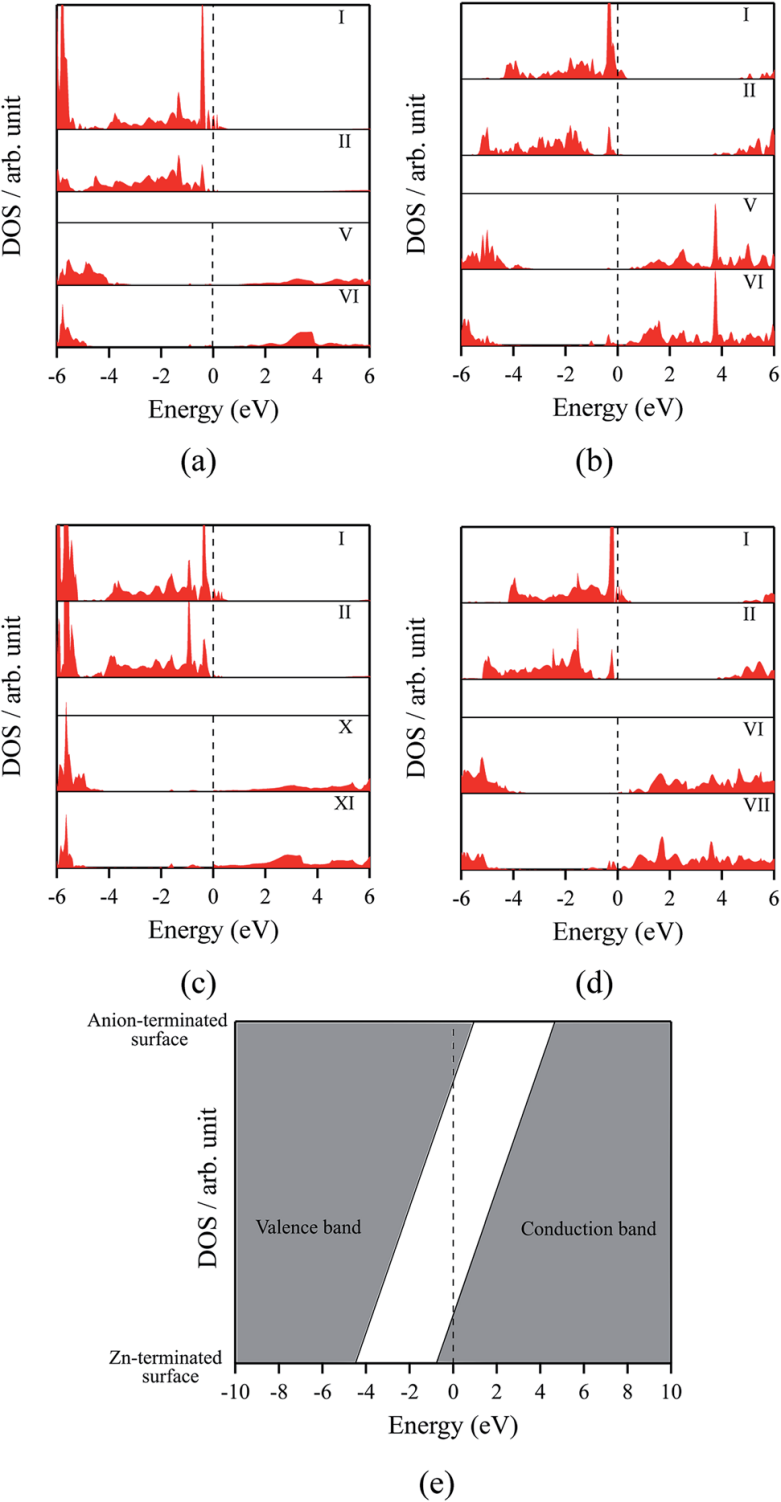
Partial density of states (PDOS) per layer for polar ZB(111) films with 6 layers for (a) ZnO and (b) ZnS. Partial density of states (PDOS) per layer for polar WZ(0001) films for (c) ZnO with 11 layers and (d) ZnS with 7 layers (the smallest thicknesses for which these structures are stable). The vertical dashed line indicates the Fermi level (e) Schematic band structure diagram of surface metallisation. A similar figure but expanded with PDOS plots for all the layers are given in the ESI.†

The polar WZ(0001) and ZB(111) films are metallic at all thicknesses found to be stable consistent with the results of Wander *et al.*^45^ and Carlsson^46^ for ZnO (see also ref. 11 and 12), with partially filled bands crossing the Fermi level around the *Γ* point (shown in Fig. 5a for the ZnO ZB(111) film with 6 layers). Fig. 6 shows the partial density of states for each layer in these polar films; these indicate that there are partially filled bands at the outermost surfaces. The main contribution at the Fermi level comes from the partially-filled anion p states in the anion-terminated surface (layer I), with also a small contribution from the Zn 4s states at the cation-terminated surface (layer VI in Fig. 6a and b/layer XI or VII in Fig. 6c and d) readily rationalised using the schematic diagram in Fig. 6e and a consideration of the electrostatic potentials in the different layers; Noguera and Goniakowski^5^ classify such behaviour as the large thickness regime for stoichiometric films in which the band gap is zero as a result of charge redistribution.

We have carried out a topological analysis of the electron charge density, performed according to Bader's AIM prescriptions,^47,48^ using the TOPOND package^49,50^ as integrated in the CRYSTAL14 code. The charge transfer in the polar surfaces is reflected in the calculated Bader charges (Table 2). In contrast, the Bader charges for the non-polar structures are similar in each layer and also to those for the three bulk phases, collected together in Table 2.

Mean Bader charges for bulk ZnO and ZnS, and for different film structures. For the films, mean values are given for atoms within the film, *i.e.*, all layers except the outermost, and also for anions and cations in the surface layers

**Table:** 

ZnO	No layers	Within film (*e*)	Mean atomic charges			
			Cation-terminated surface (*e*)		Anion-terminated surface (*e*)	
			Anion	Cation	Anion	Cation
Bulk (WZ)		±1.29	—	—	—	—
Bulk (ZB)		±1.28	—	—	—	—
Bulk (BCT)		±1.29	—	—	—	—
ZB(111)	6	±1.27	−1.27	+1.18	−1.17	+1.32
WZ(0001)	11	±1.29	−1.28	+1.15	−1.16	+1.33
Eclip. graphitic	4	±1.30	−1.28(1)	+1.28(7)	−1.28(1)	+1.28(7)
Stagg. graphitic	4	±1.28	−1.28(1)	+1.26(9)	−1.26(5)	+1.28(2)
BCT	6	±1.27	−1.29	+1.29	−1.27	+1.28
ZB(110)	6	±1.28	−1.26	+1.26	−1.26	+1.26
WZ(101̄0)	6	±1.29	−1.26	+1.27	−1.26	+1.27

a Zn atom is expelled from the surface.

**Table:** 

ZnS			Anion	Cation	Anion	Cation
Bulk (WZ)		±1.00	—	—	—	—
Bulk (ZB)		±0.99	—	—	—	—
Bulk (BCT)		±1.01	—	—	—	—
ZB(111)	6	±0.99	−0.98	+0.88	−0.84	+0.97
ZB(111)^a^	9	±1.00	−0.98	+0.80	−0.83	+0.99
WZ(0001)	7	±1.00	−1.00	+0.88	−0.85	+0.98
WZ(0001)^a^	8	±1.00	−1.00	+0.83	−0.84	+1.00
Eclip. graphitic	2	±1.00	−1.00	+1.00	−1.00	+1.00
Stagg. graphitic	3	±1.01	−1.01	+1.00	−1.00	+1.01
BCT	6	±1.01	−0.98	+0.99	−0.98	+0.99
ZB(110)	6	±1.00	−0.97	+0.98	−0.97	+0.98
WZ(101̄0)	6	±1.01	−0.97	+0.98	−0.97	+0.98

The staggered graphitic structure shows markedly different behaviour – a strong linear decrease in band gap with increasing thickness (Fig. 4a and b). At any thickness, the band gaps are lower than for all other non-polar structures.

The calculated band structures near the Fermi level for the staggered graphitic (2 and 4 layers) structure of ZnO are shown in Fig. 5b and c. Even though the band gaps of the staggered graphitic films decrease sharply with film thickness, the nature of the bands near the Fermi level remains almost the same (Fig. 5b and c). For 6 ZnO or 5 ZnS layers, the staggered graphitic structure becomes unstable with respect to ZB(111) and metallisation has clearly taken place (Fig. 5a).

The staggered graphitic structure appears at first to present a puzzle, as the sharp decrease in band gap with thickness is in stark contrast to the variations in the band gaps of the other structures in Fig. 4a and b. This structure is unique in that, while each individual plane is non-polar the bottom and top layers of the slab are not equivalent due to the staggering of the layers. If a Zn atom in the top layer has three oxygen neighbours in the same layer and a fourth oxygen atom directly below it, the Zn in the bottom layer have a different environment since they are coordinated only to three oxygens in the same layer and to no atoms in other layers. The small but significant differences in the Bader charges for atoms in the top and bottom layers in the staggered graphitic structure (Table 2) reflect this asymmetry. Thus we have formally an uncompensated polar surface in the low-thickness regime which exhibits the characteristic sharp decrease in band gap with thickness noted by Goniakowski *et al.*^6^ In the high-thickness regime, as we have seen, the staggered system changes to polar ZB(111) and the band gap is zero.

**Fig. 7:**
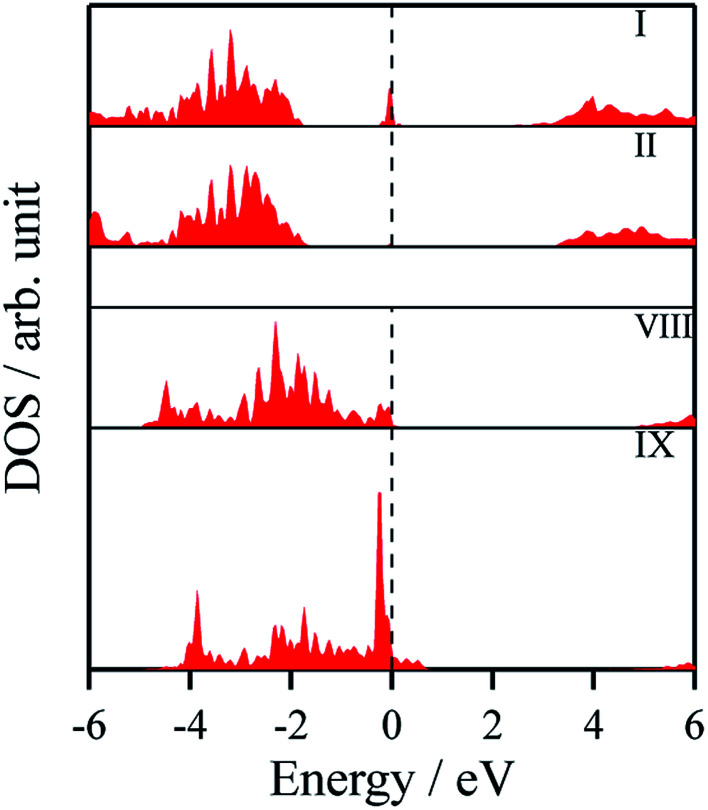
Partial density of states (PDOS) per layer for polar ZB(111) for ZnS with 9 layers. A similar figure but expanded with PDOS plots for all the layers are given in the ESI.†

The density of states and Bader charges for some of the polar surfaces of ZnS require further comment. As we have seen in Fig. 3, in some cases, WZ(0001) and ZB(111) for ≥8 layers, a Zn atom is expelled from the surface. The calculated partial density of states (PDOS) per layer for examples of such polar ZB(111) and WZ(0001) surfaces are shown in Fig. 7. Again charge transfer is highly restricted to the outer surface layers and is reflected in the contribution from these layers around the Fermi level. At the Fermi level there are contributions from the partially filled anion 3p states at the anion-terminated surface (layer IX) from just one of the four sulfur atoms, and from the Zn 3d states at the cation-terminated surface. The cation that is expelled has a much smaller Bader charge than the other cations in the Zn-terminated surface (*e.g.* for the ZB(111) film with 9 layers, the charge on the expelled Zn is +0.28, compared with +0.97 for the other Zn in the cation-terminated surface). This is the reason for the lower average cation Bader charge for the films in which Zn are expelled from the surface, shown in Table 2, compared with the thinner films where no atom leaves the surface. The remaining Zn atoms have more positive charge than in the thinner films where Zn is not expelled (*e.g.* ZB(111) film with 6 layers, Table 2).

### D. Kinetic stability

We have seen that the energies of the polar WZ(0001) and ZB(111) films are greater than the non-polar WZ(101̄0) and ZB(110) for all film thicknesses (Fig. 2). Thus on purely energetic considerations, it expected that, for thicknesses above 4 layers (ZnO) or 2 layers (ZnS), the morphology should be dominated by these non-polar surfaces. For ZnO, ref. 11 and 51 discuss the implications of the adoption of the eclipsed graphitic form by ultra-thin films for subsequent growth and suggest that its formation favours WZ(0001) films in preference to those terminated by non-polar surfaces.

The eclipsed graphitic, BCT and WZ(0001) structures all have an eclipsed AB stacking sequence; transitions between these structures should be therefore simple in the sense that they can occur by movement of the atoms only perpendicular to the *ab*-plane. For example, Morgan^16^ has demonstrated a barrierless transition for a ten-layer slab of ZnO from eclipsed graphite → BCT. No translation of layers parallel to the *ab*-plane is required, whereas this is required for a transition to the other, non-polar surfaces. Thus, it is interesting to investigate the mechanisms and energy barriers of transitions to the non-polar structures that are lowest in energy for thick films, from the structures that are most stable for thinner films. Here we only investigate transitions to WZ(101̄0), since based on geometric considerations of the atomic rearrangements involved, energy barriers for transitions to ZB(110) are expected to be higher.

**Fig. 8:**
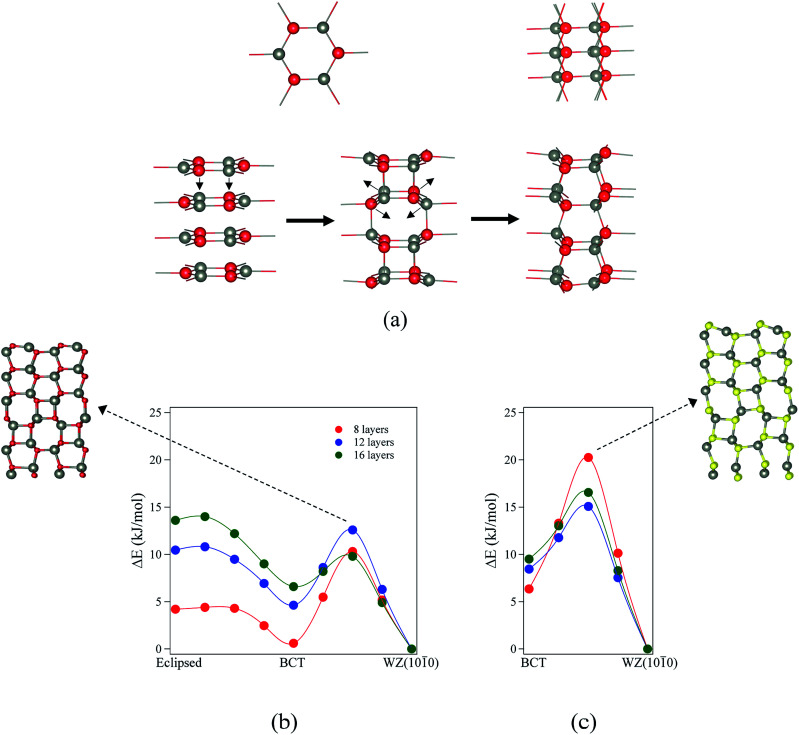
(a) Structures and indications of atomic movements required for transitions from eclipsed graphitic to WZ(101̄0) *via* a BCT structure. (b and c) Energy profiles of the structural transitions for (b) ZnO (eclipsed graphite → BCT → WZ(101̄0)) and (c) ZnS (BCT → WZ(101̄0)). All energies are relative to the final state. Zincs are grey, oxygens red, and sulfurs yellow.

**Fig. 9:**
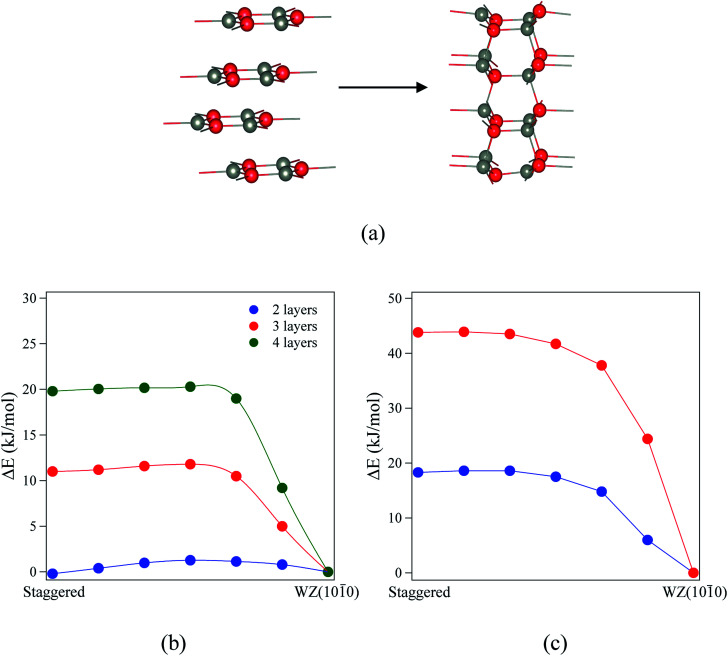
(a) Staggered graphitic and WZ(101̄0) structures. (b and c) Energy profiles of the structural transitions for (b) ZnO and (c) ZnS (staggered graphitic → WZ(101̄0)). All energies are relative to the final state. Zinc atoms are grey, oxygens red, and sulphurs yellow.

To study the transitions between structures and their kinetic stability, we have performed calculations using the DRC (dynamic reaction coordinate) methodology as implemented in CRYSTAL14. In order to calculate accurately the negative eigenvalue associated with the transition state, the SCF tolerance was set to 10^−11^ a.u.^26^ We have evaluated the energy profiles assuming a uniform variation in the lattice parameters *a* and *b* along the pathway and ignoring relaxation at these points. At the maxima these profiles revealed, we carried out a transition state search using the DRC methodology. For ZnO, we consider the transition from eclipsed graphitic to BCT and then to WZ(101̄0). As discussed previously, for ZnS films with thickness greater than 3 layers, eclipsed graphitic films spontaneously relax to the BCT structure. Thus, the transition considered for ZnS is only from BCT to WZ(101̄0). Results are shown in Fig. 8 and 9.

For ZnO, the calculated energy barriers are 0.3, 0.5 and 0.6 kJ mol^−1^ (per formula unit), for 8, 12 and 16 layers, respectively, for the eclipsed graphitic to BCT pathway; Fig. 8a shows the atomic movements involved and Fig. 8b the corresponding energy profiles; at any practical temperature the process will be effectively barrierless. For the BCT to WZ(101̄0) pathway (Fig. 8b), the energy barriers are considerably higher and decrease markedly as the number of layers increases – 9.7, 7.8 and 3.2 kJ mol^−1^ for 8, 12 and 16 layers, respectively. The low activation energy found for the movement of the atoms from the eclipsed graphitic to the BCT, and the higher barriers calculated for BCT to WZ(101̄0) path, confirm the qualitative arguments presented earlier regarding the relative ease of transitions to polar *vs.* non-polar films. For ZnS, the calculated energy barriers are 13.9, 6.6 and 7.1 kJ mol^−1^ (per formula unit), for 8, 12 and 16 layers, respectively, for the BCT to WZ(101̄0) pathway.

Finally, we consider the kinetic stability of the staggered graphitic films and we again calculate the transitions from this structure to the non-polar WZ(101̄0) films, as shown in Fig. 9a. Here the activation energies are very small or absent (Fig. 9b and c), so the transition is also effectively barrierless, except perhaps for ZnO with a thickness of 2 layers. For ZnO, the calculated energy barriers (Fig. 9b) are 1.3, 0.6 and 0.6 kJ mol^−1^ for 2, 3 and 4 layers, respectively; for ZnS, they are even smaller (Fig. 9c) 0.2 and 0.1 kJ mol^−1^ for 2 and 3 layers, respectively. These low values and thus ready formation of the non-polar WZ(101̄0) surface are in contrast with those for the eclipsed graphitic structure, reflecting the smaller movements of atoms required in the *ab*-plane.

### E. Water adsorption

Because of the considerable interest in the behaviour of water at oxide surfaces, we have considered water adsorption on some of the thin films considered, concentrating on the non-polar surfaces. We considered coverages of 25%, 50% and 100% using a 2 × 1 surface unit cell with eight layers. The adsorption energy (Δ*E*_ads_) per water molecule is given by:Δ*E*_ads_ = (*E*_a_ − *nE*_w_ − *E*_slab_)/*n*where *E*_a_ is the energy of the slab with adsorbed water, *E*_w_ the energy of an optimised isolated water molecule, *E*_slab_ the energy of the optimised isolated slab without adsorbed water, and *n* is the number of water molecules per surface unit cell. Results are corrected for the basis set superposition error BSSE using the counterpoise method.^52^

**Fig. 10:**
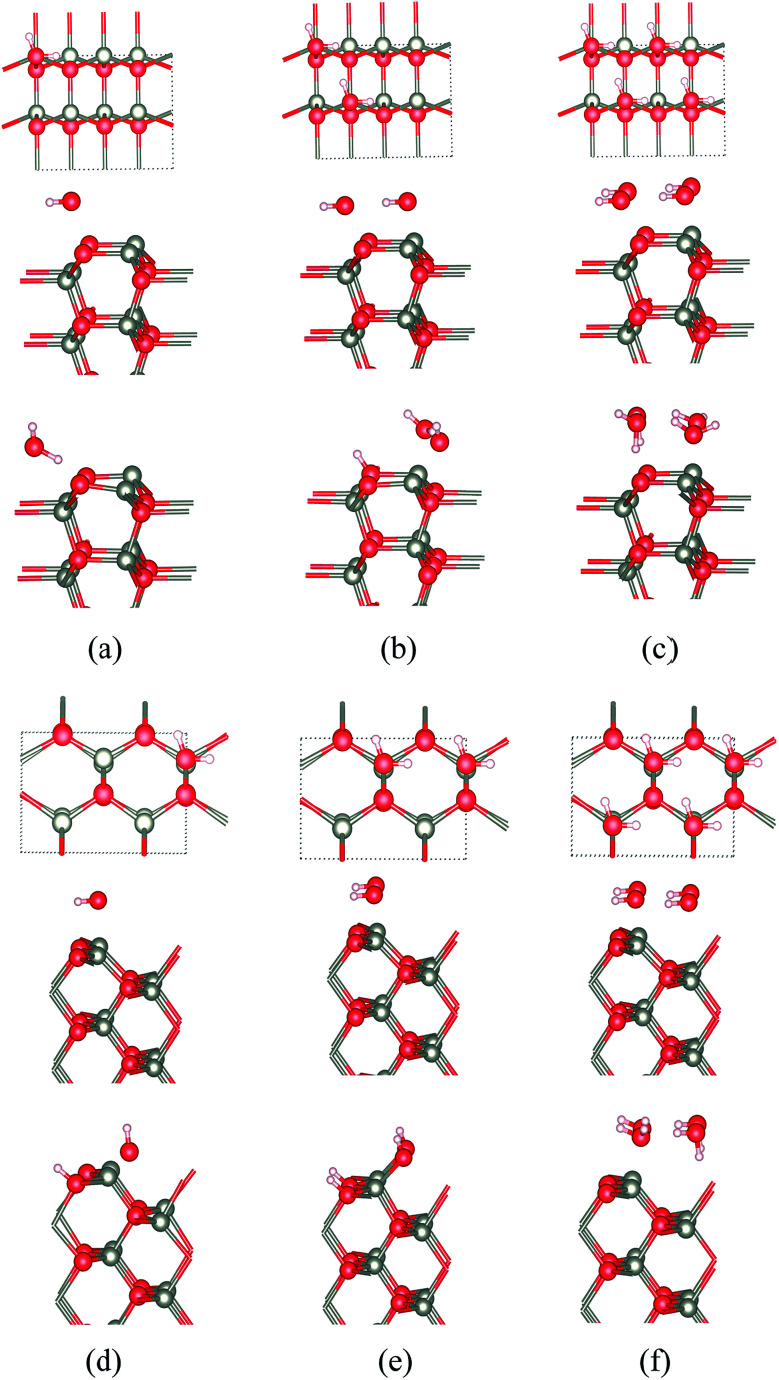
Top and side views for the initial geometries, and side views of the final geometries, for WZ(101̄0) films of ZnO with (a) 25%, (b) 50% and (c) 100% water coverage. Top and side views of ZB(110) for ZnO with (d) 25%, (e) 50% and (f) 100% water coverage. In (b), half of the water molecules dissociate. In (d and e), the water dissociates forming H^+^ and OH^−^. In (a, c and f), there is molecular adsorption.

The calculated adsorption energies for the different cases for ZnO and ZnS are shown in Table 3 and the corresponding optimised structures for ZnO shown in Fig. 10a–f. Our results agree with previous calculations for bulk ZnO,^53–55^ which conclude that on WZ(101̄0) half of the water molecules dissociate into OH^−^ and H^+^ when the surface coverage is 50% (*i.e.*, two water molecules per surface unit cell) independent of film thickness (Fig. 10b). When the coverage is 25% or 100% there is no dissociation. Such overall change with coverage is unusual and there is an apparent discrepancy here with a very recent experimental study which reports a fully dissociated monolayer.^56^

**Table:** Adsorption energies of water (in units of eV per H_2_O) on ZnO and ZnS thin films with 4 layers. M: molecular adsorption; D: water dissociation

Surface type	Coverage (%)	ZnO		ZnS	
		Δ*E*_A_ (eV)	Mechanism	Δ*E*_A_ (eV)	Mechanism
WZ(101̄0)	25	−1.50	M	−0.93	M
	50	−1.66	50% D	−0.85	M
	100	−1.35	M	−0.90	M
ZB(110)	25	−1.80	D	−1.01	M
	50	−1.62	D	−0.92	M
	100	−1.17	M	−0.88	M
BCT	25	−0.82	M	−0.82	M
	50	−1.04	M	−0.83	M
	100	−1.19	M	−0.89	M

At the ZB(110) surface of ZnO, water molecules are fully dissociated into OH^−^ and H^+^ when the surface coverages are 25% and 50%, as shown in Fig. 10. This intriguing difference in behaviour does not appear to have been reported previously and merits future experimental and theoretical study. As for WZ(101̄0), when the coverage is 100%, there is no water dissociation. We have not observed water dissociation at the surface of BCT films for any coverage.

For ZnS, we have not observed water dissociation at any surface and the adsorption energies are smaller than for ZnO (Table 3).

Calculations of water adsorption on the eclipsed and staggered graphitic films were also attempted. However, in the presence of adsorbed water, these structures are not stable. Eclipsed graphitic nanofilms transform into BCT films and staggered graphitic nanofilms transform into WZ(101̄0) films, consistent with the low energy barriers we have noted earlier for such structural transitions.

## Conclusions

We have predicted, from analysis of the energy landscapes of ultra-thin nanofilms of ZnO and ZnS calculated using periodic hybrid density functional theory, new minima corresponding to graphitic structures in which the layers are not eclipsed but staggered with respect to each other. These show a spontaneous symmetry breaking across the film accompanied by a sharp reduction in the band gap with thickness, associated with the transfer of electron density from one layer to another and the asymmetry of this particular structure.

For polar surfaces the complexity of the energy landscape is due to the many different mechanisms which operate to remove or reduce surface dipoles depending on nanofilm thickness. The formation of the BCT structure is kinetically favoured for some layer thicknesses while the eclipsed and staggered graphitic films become less kinetically stable as the number of layers increases. There are significant barriers to the formation of the non-polar WZ(101̄0) from the eclipsed graphitic structure, which suggests why polar surfaces are observed experimentally.

For ZnS overall the low-energy polymorphism is less rich than for ZnO, reflecting the relative preferences of S and O for 4- *vs.* 3-fold planar coordination. We predict spontaneous loss of zinc atoms from the polar ZnS nanofilms, but not the polar ZnO analogues or any non-polar film.

Our results for water adsorption on the nanofilms predict that while there is no dissociation on any ZnS film, for ZnO dissociation into OH^−^ and H^+^ takes place not only on (101̄0) würtzite, but also on (110) zinc blende.

While we have concentrated on ZnO and ZnS in this paper, we anticipate similar energy landscapes and structures for thin films of oxides and sulphides which adopt the würtzite or zinc blende structure in the bulk.

## Conflicts of interest

There are no conflicts to declare.

## Supplementary Material

NA-001-C8NA00155C-s001
