# Supplementary material for: Graphene and novel graphitic ZnO and ZnS nanofilms: the energy landscape, non-stoichiometry and water dissociation
Source: Nanoscale Adv. 2019 Apr 1;1(5):1924–35. doi: 10.1039/c8na00155c (PMC9419858; doi:10.1039/c8na00155c)
Supplement: NA-001-C8NA00155C-s001 [file NA-001-C8NA00155C-s001.pdf]

Electronic Supplementary Material (ESI) for Nanoscale Advances.
This journal is © The Royal Society of Chemistry 2019

Supporting information

Graphene and novel graphitic ZnO and ZnS nanofilms: the energy landscape, non-stoichiometry and water dissociation

Sergio Conejeros*[a,b], Neil L. Allan[a], Frederik Claeyssens[c] and Judy N. Hart[d]

[a]School of Chemistry, University of Bristol, Cantock's Close, Bristol, U.K., BS8 1TS.

[b]Departamento de Química, Universidad Católica del Norte, Av. Angamos 0610, Antofagasta, Chile, 124000.

[c]Department of Engineering Materials, University of Sheffield, Sir Robert Hadfield Building, Mappin Street, Sheffield S1 3JD

[d]School of Materials Science & Engineering, UNSW Sydney, NSW 2052 Australia

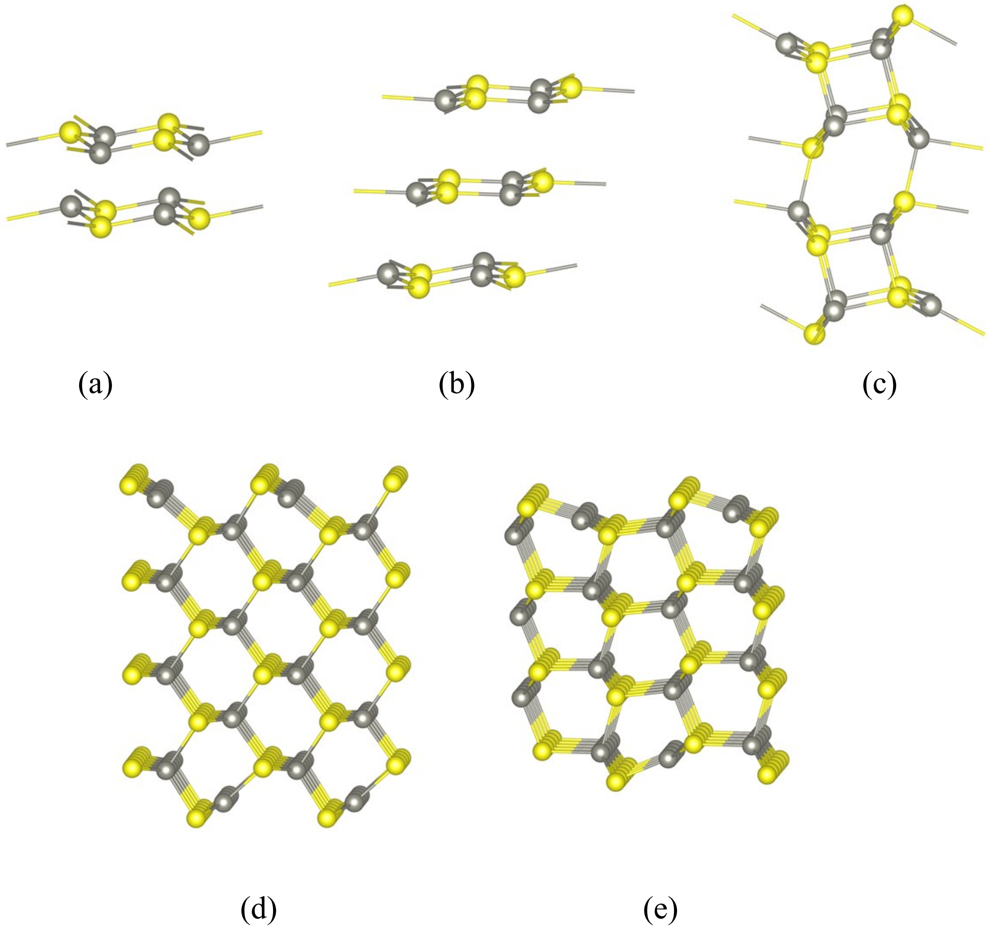

Figure S1. Optimised structures of those ZnS films where there is substantial structural relaxation from the ideal structures shown in Figure 1: a) Eclipsed graphitic (2 layers), b) Staggered graphitic (3 layers), c) non-polar BCT, d) non-polar ZB(110) and e) non-polar WZ(10$\bar{1}$0). Zn atoms are grey, S yellow.

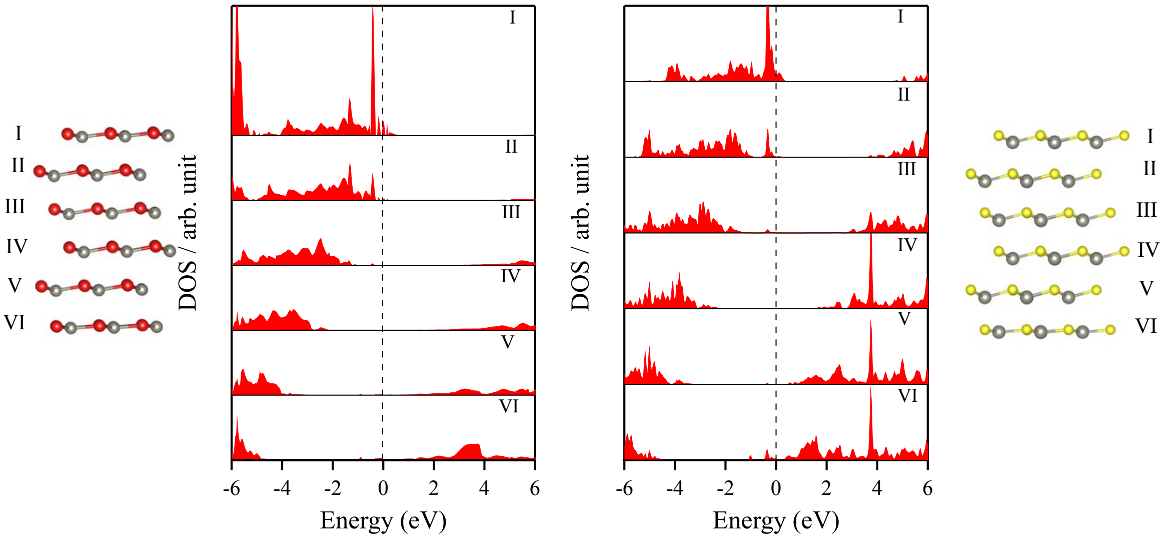

Figure S2. Partial density of states (PDOS) per layer for polar ZB(111) films with 6 layers for a) ZnO and b) ZnS. Corresponding structures are shown beside the DOS. The vertical dashed line indicates the Fermi level.

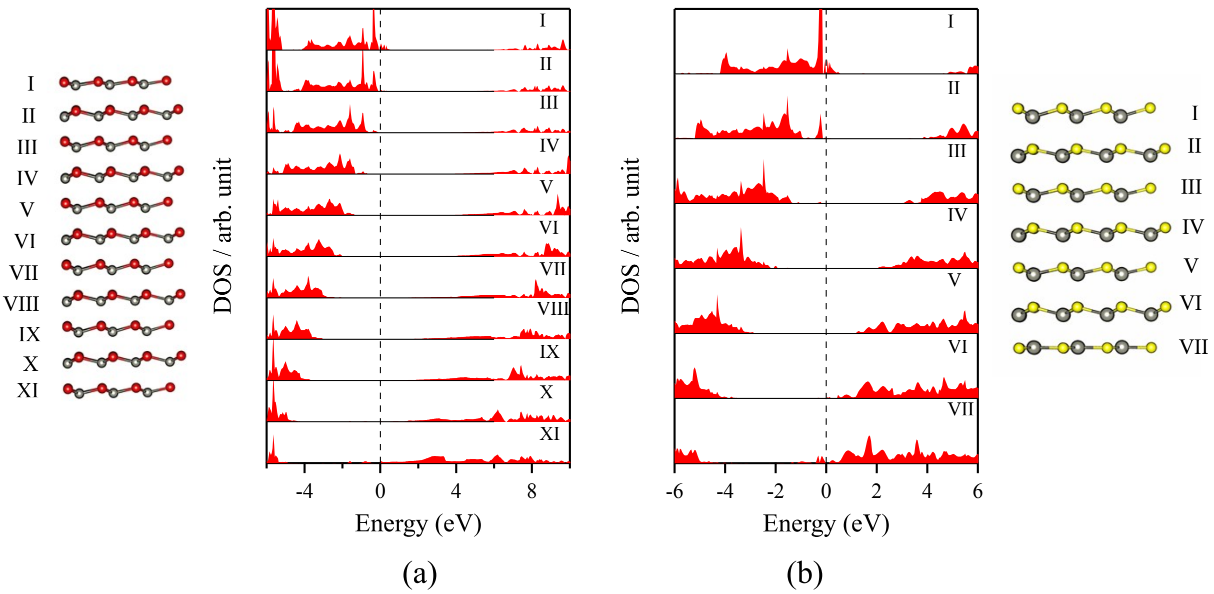

Figure S3. Partial density of states (PDOS) per layer for polar WZ(0001) films for a) ZnO with 11 layers and b) ZnS with 7 layers (the smallest thicknesses for which these structures are stable). Corresponding structures are shown beside the DOS. The vertical dashed line indicates the Fermi level.

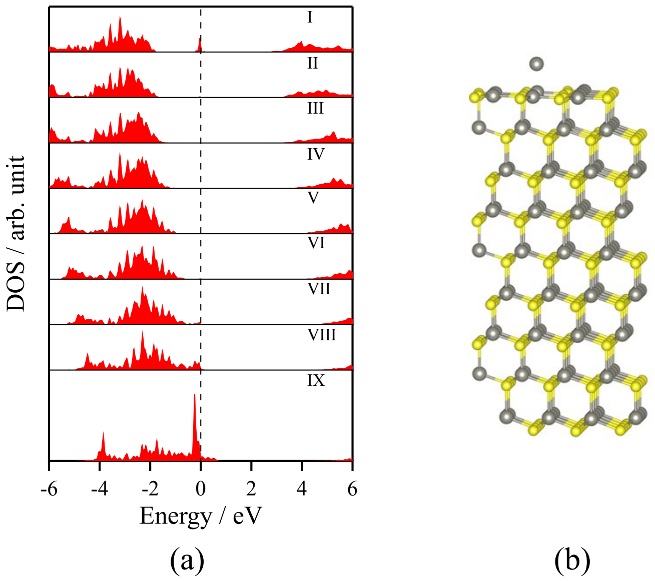

Figure S4. Partial density of states (PDOS) per layer for polar ZB(111) for ZnS with 9 layers.
